# Supplementary material for: Spatio-temporal development of cuticular ridges on leaf surfaces of Hevea brasiliensis alters insect attachment
Source: R Soc Open Sci. 2020 Nov 4;7(11):201319. doi: 10.1098/rsos.201319 (PMC7735362; doi:10.1098/rsos.201319)
Supplement: Supplementary information - table and figures [file rsos201319supp1.docx]

**Spatio-temporal development of cuticular ridges on leaf surfaces of *Hevea brasiliensis* alters insect attachment**

*Venkata A. Surapaneni, Georg Bold, Thomas Speck, Marc Thielen*

**Supplementary information**

**Table S1.** Age, colour and surface properties of the adaxial leaves of *Hevea brasiliensis* at different ontogenetic stages

**Table S2.** Median values of the roughness parameters from the replicas of leaves at stages S2B and S3 (on which traction experiments were conducted) with contaminated negative moulds. The roughness values underestimate the real values given in Table 1 in the main manuscript.

**Figure S1.** *Leaf growth versus leaf stage:* The image shows the length of midrib (growth) versus leaf age for four *Hevea brasiliensis* leaves

**Figure S2.** Schematic of the experimental set up used for insect walking experiments to measure maximum traction forces. PC – Computer, AD – Amplifier, FT – Force transducer, L – Light source, W – Molten beeswax, B – Beetle, R – Replica and H – Human hair.

**Figure S3.** 3D reconstruction of CLSM observations of replicas of leaves at different stages using Mountains Map Premium ver. 7: (a) Stage 1 (b) Stage 2A (c) Stage 2B (d) Stage 3 (e) Stage 2B (leaf remnants attached) (f) Stage 3 (leaf remnants attached) (g) Stage 4 (h) Stage 5.

**Figure S4.** Leaf cuticle patch attached to the epoxy replica (a) Leaf cuticle (b) Epoxy

**Figure S5.** *Remains of plant material on replicas:* Except for a few patches (as shown in Fig. 2 and Fig. 3), the entire area of the epoxy replicas of leaves at stages S2B and S3 retained plant cuticular material, even after KOH treatment. (a - b) CLSM images of positive replicas of leaves at stages (a) S2B and (b) S3. (c) CLSM image of a region much closer to the base of the leaf replica at transition stage S3 in which the ridge morphology is much more similar to that of adult stages (Fig. 3).

**Figure S6.** *Insect traction forces:* Box plot showing the differences in traction forces of *Leptinotarsa decemlineata (n=40)* for a set of statistical replicates of PDMS replica surfaces of leaves at various growth stages compared with glass and PDMS glass replicas. The traction force values for stage 2B and stage 3 could only be calculated from replicas from contaminated moulds (box plots in grey) and therefore overestimate the real values (see Results section).

**Figure S7.** Correlation plot of log transformed values of mean insect traction forces versus mean aspect ratio of the ridges taken over each replicate (without data from stages S2B and S3). Pearson’s test showed strong correlation of insect traction forces with mean aspect ratio of the ridges with *R = -0.91*

**Table S1.** Age, colour and surface properties of the adaxial leaves of *Hevea brasiliensis* at different ontogenetic stages

| **Leaf Stage** | **Notation** | **Leaf Age** | **Leaf colour** | **Surface structure** |
| --- | --- | --- | --- | --- |
| Stage 1 | S1 | 13 ± 2 | Shiny brown | Smooth cells |
| Stage 2 (apical) | S2A | 15 ± 3 | Shiny brown | Smooth cells |
| Stage 2 (basal) | S2B | 15 ± 3 | Pale green | Ridges  (high aspect ratio) |
| Stage 3 | S3 | 16 ± 3 | Pale green | Ridges  (high aspect ratio) |
| Stage 4 | S4 | 21 ± 4 | Pale green | Ridges  (labyrinth type) |
| Stage 5 | S5 | > 60 | Dark green | Ridges  (labyrinth type) |

**Table S2.** Median values of the roughness parameters from the replicas of leaves at stages S2B and S3 (on which traction experiments were conducted) with contaminated negative moulds. The roughness values underestimate the real values given in Table 1 in the main manuscript.

| **Surface** | **Age (days)** | ***Ra* (µm)** | ***Rc* (µm)** | ***Rsm* (µm)** | **Aspect ratio = *Rc/Rsm*** | ***Rsk*** |
| --- | --- | --- | --- | --- | --- | --- |
| S2B | 15 ± 3 | 0.044 | 0.152 | 1.465 | 0.102 | -0.48 |
| S3 | 16 ± 3 | 0.053 | 0.173 | 1.33 | 0.129 | -0.495 |

**
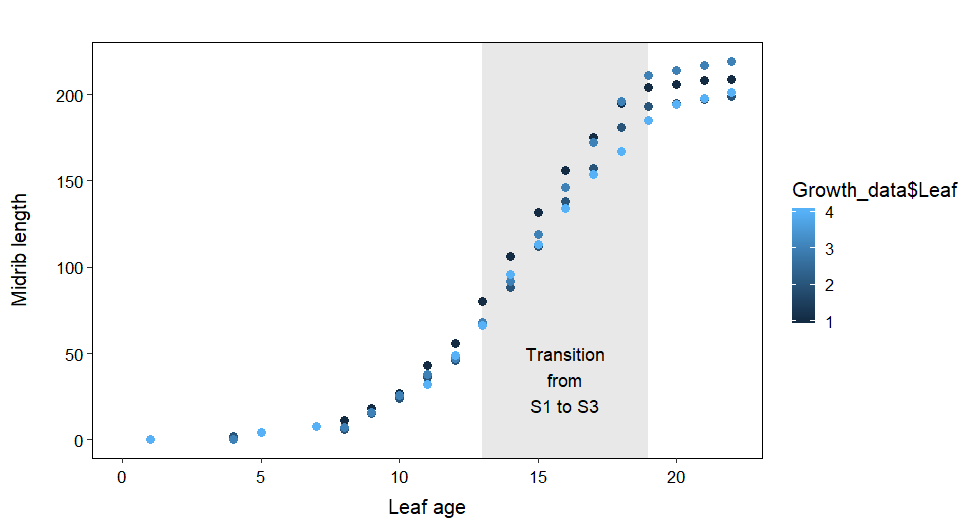
**

**Figure S1.** *Leaf growth versus leaf age:* The image shows the length of midrib (growth) versus leaf age for four *Hevea brasiliensis* leaves


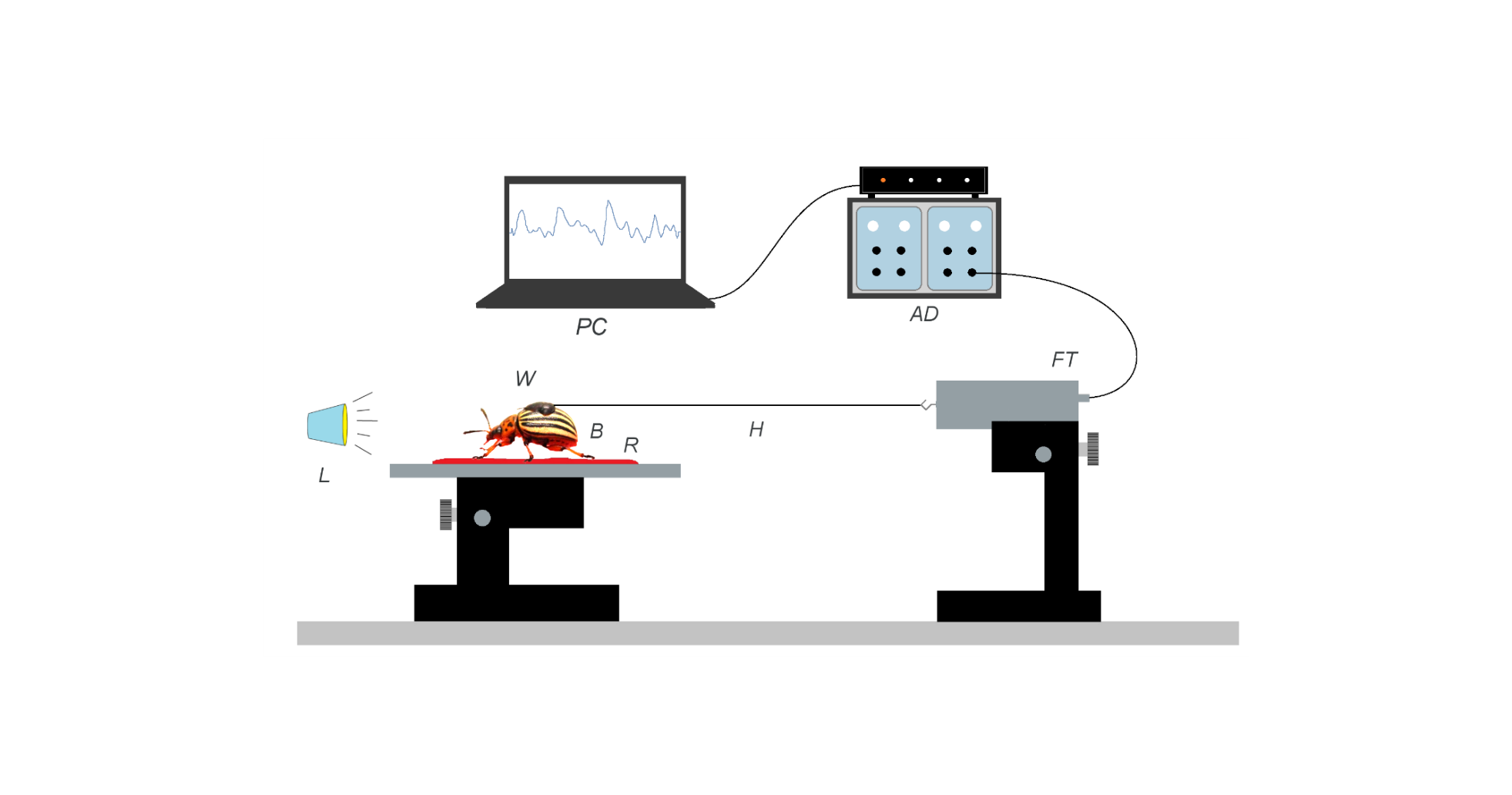


**Figure S2**. Schematic of the experimental set up used for insect walking experiments to measure maximum traction forces. PC – Computer, AD – Amplifier, FT – Force transducer, L – Light source, W – Molten beeswax, B – Beetle, R – Replica and H – Human hair.


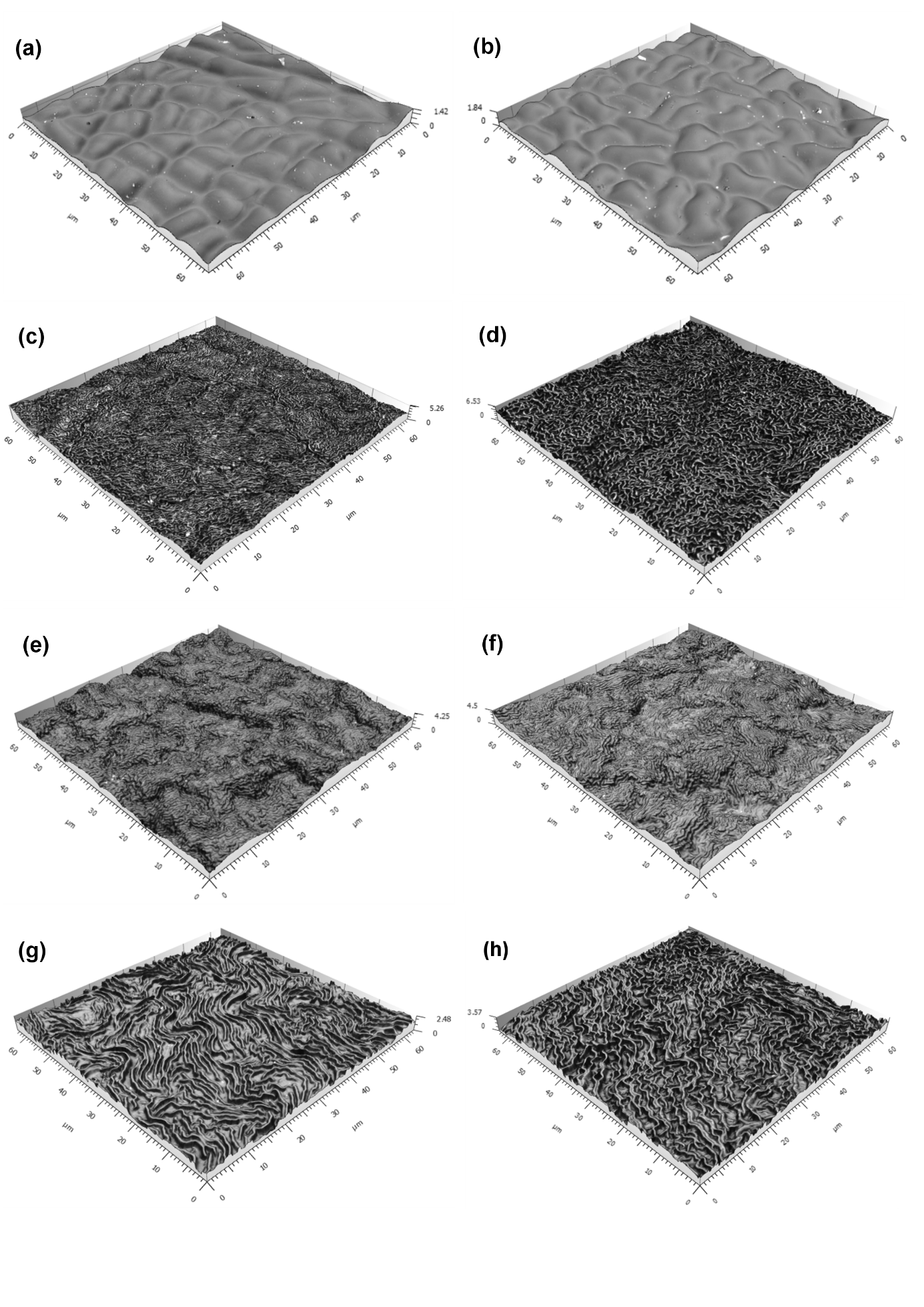


**Figure S3.** 3D reconstruction of CLSM observations of replicas of leaves at different stages using Mountains Map Premium ver. 7: (a) Stage 1 (b) Stage 2A (c) Stage 2B (d) Stage 3 (e) Stage 2B (leaf remnants attached) (f) Stage 3 (leaf remnants attached) (g) Stage 4 (h) Stage 5.


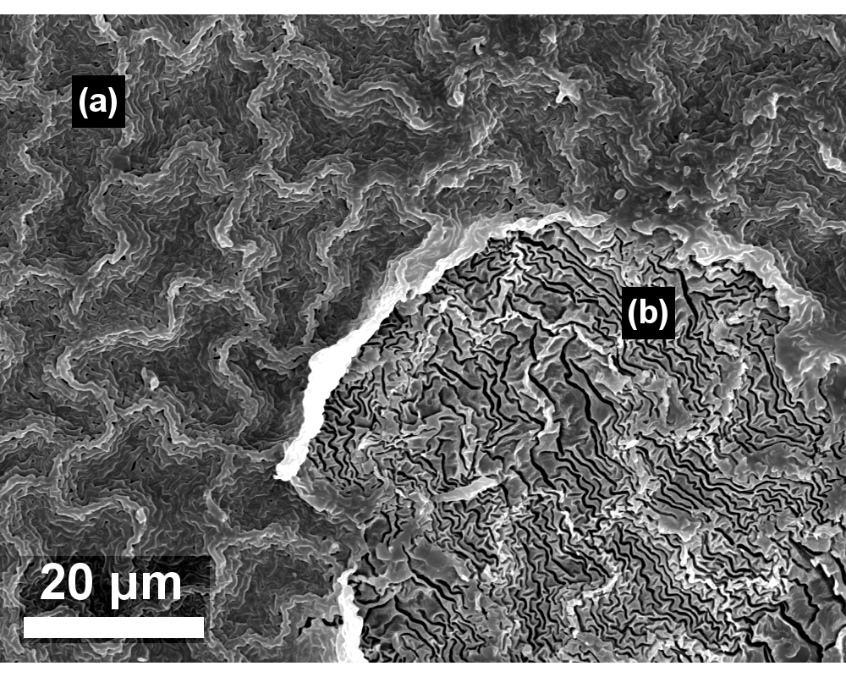


**Figure S4.** Leaf cuticle material attached to the (negative) epoxy replica (a) Leaf cuticle (b) Epoxy

**
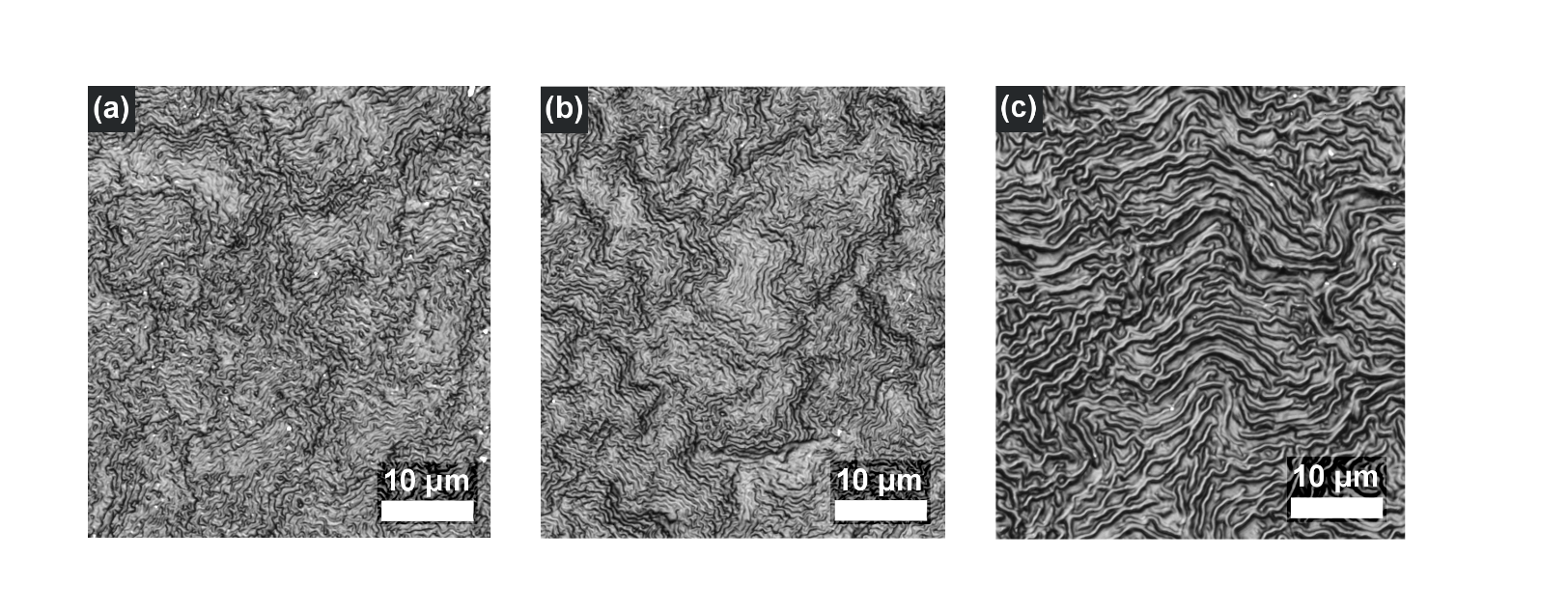
**

**Figure S5.** *Remains of plant material on replicas:* Except for a few patches (as shown in Fig. 2 and Fig. 3 in the main manuscript), the entire area of the epoxy replicas of leaves at stages S2B and S3 retained plant cuticular material, even after KOH treatment. (a - b) CLSM images of positive replicas of leaves at stages (a) S2B and (b) S3. (c) CLSM image of a region much closer to the base of the leaf replica at transition stage S3 in which the ridge morphology is much more similar to that of adult stages (Fig. 3 in the main manuscript).

**
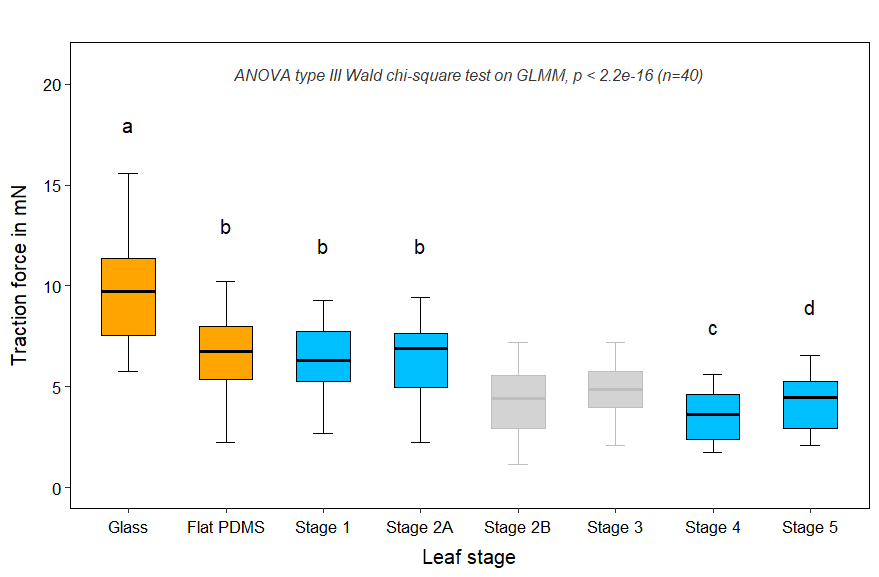
Figure S6.** *Insect traction forces:* Box plot showing the differences in traction forces of *Leptinotarsa decemlineata (n=40)* for a set of statistical replicates of PDMS replica surfaces of leaves at various growth stages compared with glass and PDMS glass replicas. The traction force values for stage 2B and stage 3 could only be calculated from replicas from contaminated moulds (box plots in grey) and therefore overestimate the real values (see Results section).

**
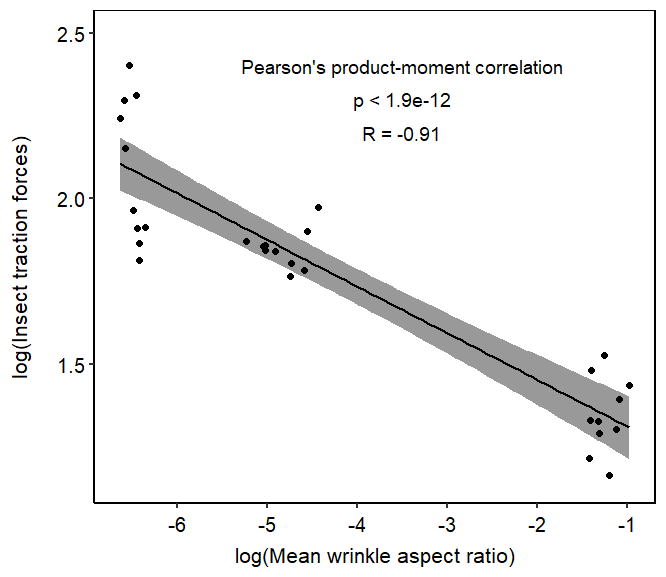
**

**Figure S7.** Correlation plot of log transformed values of mean insect traction forces versus mean aspect ratio of the ridges taken over each replicate (without data from stages S2B and S3). Pearson’s test showed strong correlation of insect traction forces with mean aspect ratio of the ridges with *R = -0.91*
